# Clear Aligner Therapy Assisted by Implant Anchorage and S‐Shaped Root Control Attachments for Anterior Open Bite Correction: A 30‐Month Treatment Case Report

**DOI:** 10.1155/crid/7446066

**Published:** 2026-09-07

**Authors:** Yaqiu Chen, Yewei Xin, Chang Liu, Xiaojing Sun, Juanying Yang, Qian Jiang, Feng Qiu

**Affiliations:** ^1^ The Department of Stomatology, Liuzhou Workers′ Hospital, Liuzhou, China; ^2^ The Department of Orthodontics, Affiliated Stomatology Hospital of Guilin Medical University, Guilin, China; ^3^ The Department of Orthodontics, Stomatology Hospital of Guilin, Guilin, China; ^4^ The Department of Maxillofacial Surgery, Affiliated Stomatology Hospital of Guilin Medical University, Guilin, China

**Keywords:** anterior open bite, clear aligner, miniscrew implant, S-shaped root control

## Abstract

Anterior open bite is a prevalent malocclusion that can cause occlusal dysfunction and markedly reduce masticatory efficiency. This case report describes the successful management of a 37‐year‐old female with an anterior open bite secondary to excessive vertical development in the molar region and clockwise mandibular rotation. The treatment combined clear aligners with implant anchorage for molar intrusion, alongside S‐shaped double‐arm root control attachments to facilitate the uprighting of severely tilted teeth. Following the placement of four implants in the maxillary posterior region, the therapy was systematically carried out in four distinct phases: molar intrusion, premolar intrusion, correction of mandibular anterior tooth axial inclination, and final occlusal refinement. After 30 months, both the occlusal relationship and facial profile were significantly improved, with complete resolution of the anterior open bite and establishment of normal overbite and overjet. Cephalometric analysis confirmed favorable outcomes, including counterclockwise rotation of the mandible. This case highlights the efficacy of an implant‐assisted clear aligner protocol augmented by specialized attachments for managing complex anterior open bite, thereby expanding the scope of clear aligner therapy.

## 1. Introduction

Anterior open bite (AOB) poses a significant clinical challenge, characterized by a lack of vertical overlap between the maxillary and mandibular incisors when the posterior teeth are in occlusion. This condition can considerably impair masticatory function, speech articulation, and facial esthetics [1, 2]. The etiology of AOB is multifactorial, encompassing skeletal components (e.g., excessive posterior alveolar vertical growth, increased mandibular plane angle, and clockwise mandibular rotation), dental factors (e.g., abnormal eruption patterns), and functional habits (e.g., tongue thrusting and abnormal swallowing) [3, 4]. Notably, cases primarily driven by excessive posterior vertical development and consequent clockwise mandibular rotation are among the most complex to correct orthodontically.

Conventional orthodontic treatment with fixed appliances, although effective in many cases, is often associated with compromised esthetics, patient discomfort, and challenges in maintaining oral hygiene [5]. Furthermore, it often struggles to achieve true molar intrusion—a key objective in AOB correction—due to the lack of absolute anchorage, which can result in unstable outcomes [6]. Although orthognathic surgery offers a definitive skeletal correction, it entails substantial risks, prolonged recovery period, and significant financial cost [7]. Clear aligner therapy (CAT) has emerged as a popular alternative that addresses several limitations of fixed appliances by offering superior esthetics, enhanced comfort, and improved oral hygiene access [8]. However, CAT introduces its own set of difficulties in achieving complex tooth movements. Specifically, predictable posterior intrusion and the correction of severe axial inclinations remain challenging due to the biomechanical limitations of thermoplastic materials [9, 10]. Although the “bite block effect” of aligners has been suggested as a mechanism for posterior intrusion, its consistent clinical efficacy has not been firmly established [11, 12].

The advent of temporary anchorage devices (TADs) has markedly advanced orthodontic capabilities by providing absolute anchorage, enabling controlled and predictable application of forces for tooth movements that were once difficult to accomplish [13, 14]. Integrating TADs with CAT opens new avenues for addressing these biomechanical limitations. Furthermore, the development of specialized attachments, such as the novel S‐shaped root control attachments (SRCAs), provides enhanced control over root position in cases of severe inclination. This case report illustrates a novel protocol that synergistically combines implant‐assisted anchorage with SRCAs in CAT to correct a skeletal AOB resulting from excessive posterior vertical development, demonstrating the potential of this integrated approach for managing complex malocclusions.

## 2. Case Presentation

This case report was prepared in accordance with the Joanna Briggs Institute (JBI) critical appraisal checklist for case reports to ensure completeness and transparency of reporting.

### 2.1. Diagnosis and Etiology

A 37‐year‐old female presented with the chief complaint of an AOB. The patient reported no temporomandibular joint symptoms and no significant family or genetic history of malocclusion. She was systemically healthy.

**Figure 1:**
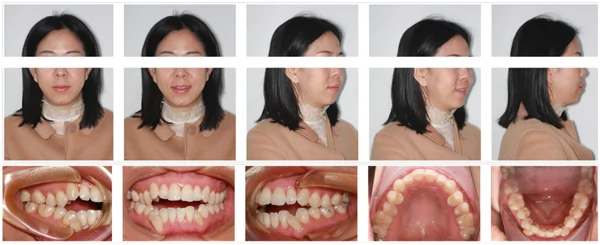
Initial facial and intraoral photographs.

Initial facial photographs (Figure 1) revealed a convex facial profile, a retrognathic chin, protrusive upper and lower lips, increased lower facial height, and dental midlines that were deviated from the facial midline.

Intraoral examination (Figure 1) confirmed a permanent dentition with a moderate standard of oral hygiene and the absence of Tooth 28. The occlusal assessment indicated bilateral mesial molar relationships, a left Class III canine relationship, and a right Class II canine relationship. An AOB was evident, accompanied by mild (2 mm) maxillary crowding and severe (9 mm) mandibular crowding. Tooth 33 was notably rotated, and tooth 43 was positioned entirely outside the dental arch. The mandibular dental midline was deviated 3 mm to the right, and a locked occlusion was observed between Teeth 15 and 46.

A comprehensive orofacial functional assessment was performed prior to treatment planning. Evaluation of tongue posture at rest, tongue thrust during swallowing, and overall swallowing patterns revealed no abnormal functional habits or orofacial dysfunction. In the absence of contributory myofunctional factors, referral for myofunctional therapy was not indicated, and the etiology was attributed primarily to skeletal factors.

**Figure 2:**
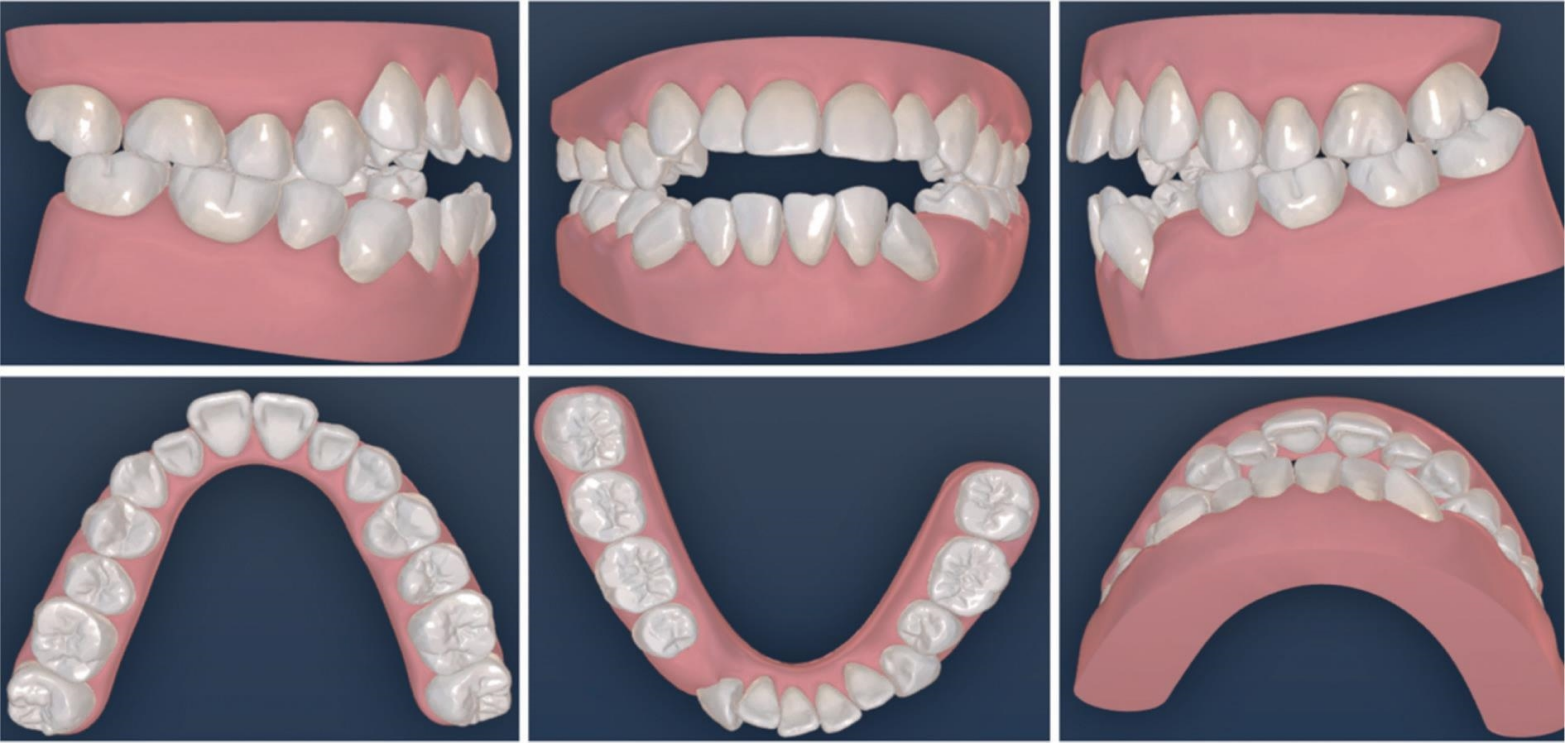
Pretreatment digital model.

Evaluation of the digital models (Figure 2) corroborated the crowding assessments and further detailed the malocclusion, showing a curve of Spee with a mean depth of 0.9 mm, an AOB of 6 mm, an overjet of 2 mm, and Bolton ratios of 85.24% (anterior) and 94.57% (overall).

**Figure 3:**
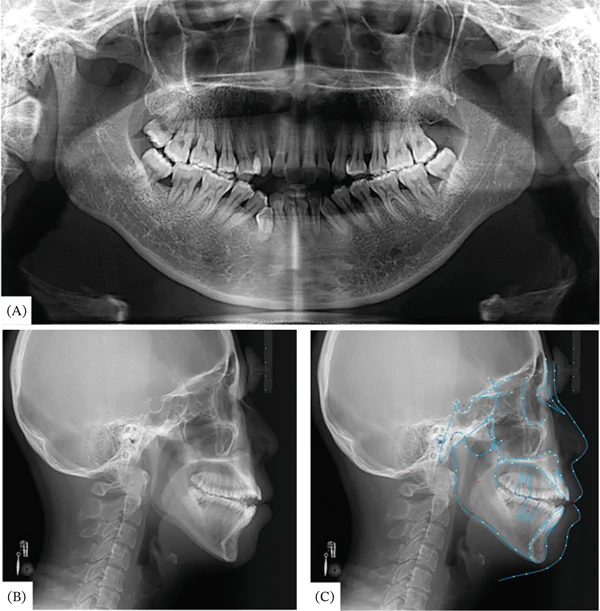
Pretreatment radiographs. (A) Panoramic radiograph, (B) lateral cephalogram, and (C) lateral cephalogram tracing.

Radiographic examination provided additional insights. The panoramic radiograph (Figure 3A) revealed slightly asymmetric condylar morphology, mild alveolar bone resorption, and confirmed the congenital absence of Tooth 28. Lateral cephalometric analysis (Figure 3B,C) identified a skeletal Class II relationship (SNA: 82.1°; SNB: 75.61°; ANB: 6.49°) characterized by mandibular retrognathism and a hyperdivergent growth pattern (MP‐SN: 50.63°). The maxillary incisors demonstrated retroclination (U1‐SN: 100.91°), whereas the inclination of the mandibular incisors was within normal limits.

### 2.2. Treatment Alternatives

Two distinct treatment options were formulated and presented to the patient based on her diagnostic records.

**Figure 4:**
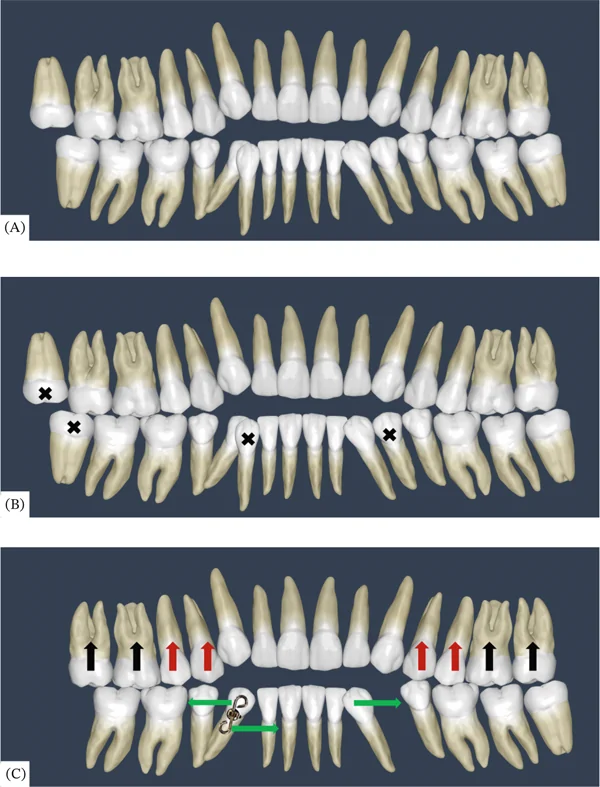
Treatment plan: (A) schematic diagram before dentition treatment; (B) Selection of tooth extraction location; and (C) Schematic diagram of tooth movement. Black arrow: vertical intrusion of molars. Red arrow: vertical intrusion of the premolars. Green arrows: mesial‐distal traction of the right mandibular premolar and maxillary canines and uprighting of the right mandibular premolars.

**Figure 5:**
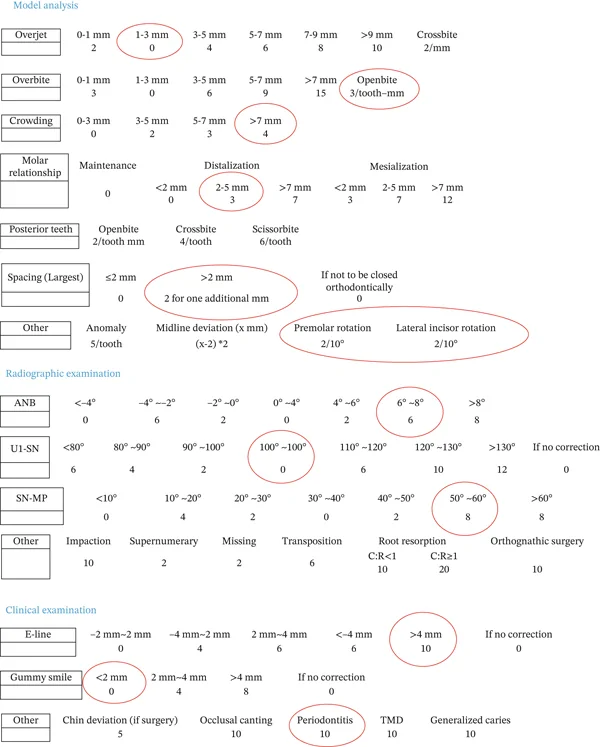
The CAT‐CAT score of first option is 89.134.

The first option involved comprehensive orthodontic treatment with clear aligners, accompanied by a strategic extraction plan. This approach required the extraction of four teeth: Teeth 18, 28, 34, and 43. The rationale for this specific selection was twofold. In the maxilla, the removal of the third molars (Teeth 18 and 28) was intended to utilize their wedge effect, thereby reducing posterior occlusal height to aid in open bite correction while simultaneously resolving the mild arch crowding. In the mandible, the decision to extract Tooth 43 rather than the adjacent first premolar (Tooth 44) was guided by a comprehensive evaluation integrating malposition severity, periodontal status, functional requirements, and esthetic outcomes. With regard to malposition severity, Tooth 43 was positioned entirely outside the dental arch with a severe mesial inclination exceeding 30 degrees; its orthodontic correction via CAT alone would have required unpredictable crown‐and‐root movements over an extended distance, substantially increasing treatment complexity and compromising predictability. Tooth 44, although inclined mesially, remained within the alveolar envelope and was accessible for controlled torque correction using specialized attachments. From a periodontal perspective, the severely ectopic position of Tooth 43 was associated with inadequate buccal alveolar bone coverage—heightening the risk of dehiscence and fenestration during orthodontic force application in an already periodontally compromised patient—whereas Tooth 44 demonstrated more favorable bilateral bone support. Functionally, the first premolar, when uprighted and appropriately torqued into the canine position, can provide adequate cusp guidance in lateral excursions; this was verified in the digital treatment simulation prior to treatment commencement. Esthetically, the crown morphology of Tooth 44 following controlled axial correction was determined to be compatible with the anticipated smile arc and facial contour, as confirmed by pretreatment visualization in the Smartee planning software. This strategic extraction decision significantly reduced overall biomechanical complexity and enhanced treatment predictability, as reflected by the treatment complexity assessment. The treatment would then proceed with dentition alignment and AOB correction through posterior intrusion. The Clear Aligner Treatment Complexity Assessment Tool (CAT‐CAT) score for this option was 89.134, indicating moderate complexity (Figure 4). The planned extraction design and tooth movement vectors of this treatment plan are demonstrated in Figure 5.

The second option was a combined orthodontic–orthognathic surgical approach. This comprehensive strategy included presurgical orthodontic decompensation and alignment, followed by orthognathic surgery. The surgical procedure was planned as a maxillary Le Fort I segmental osteotomy combined with a mandibular bilateral sagittal split ramus osteotomy, with a genioplasty on standby. A final phase of postsurgical orthodontic refinement was planned to idealize the occlusal outcome. The patient was thoroughly informed about the inherent surgical risks, the extended treatment duration, and the considerable cost associated with this option.

Both alternatives were designed to resolve the dentitional crowding and correct the AOB. Crowding would be managed through the utilization of extraction spaces, whereas the AOB would be addressed via a combination of posterior tooth intrusion and anterior tooth extrusion. The surgical option, however, offered the advantage of definitive skeletal reconstruction through multidisciplinary collaboration. After detailed discussion and providing informed consent, the patient explicitly refused the surgical intervention, expressing a strong preference for a nonsurgical, esthetic, and comfortable treatment. Consequently, the first option—nonsurgical treatment with clear aligners—was selected.

### 2.3. Treatment Goals

The treatment objectives were as follows:

• To resolve dentitional crowding and establish proper arch alignment.
• To achieve maximum possible posterior intrusion to induce counterclockwise mandibular rotation.
• To correct the AOB and establish normal overbite and overjet relationships.
• To improve the axial inclination of severely tilted teeth within biological and biomechanical limits.
• To enhance the facial profile and soft tissue esthetics.
• To maintain periodontal health throughout the treatment.

**Figure 6:**
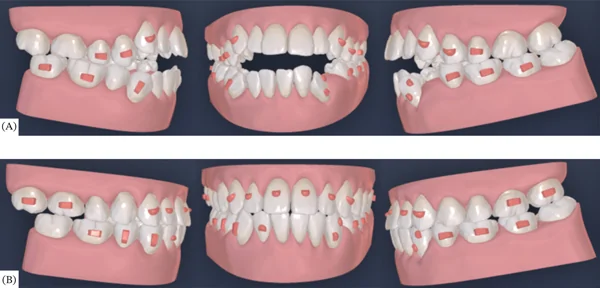
Dental changes as seen in the Smartee software: (A) pretreatment and (B) posttreatment.

The comprehensive treatment plan and anticipated dental changes were visualized using Smartee treatment planning software, which demonstrated the expected transformation from the initial malocclusion to the desired final occlusion (Figure 6).

### 2.4. Treatment Process

According to the staged treatment plan, the treatment was systematically divided into four phases: (1) intrusion of maxillary molars, (2) intrusion of maxillary premolars, (3) correction of the axial inclination of mandibular anterior teeth, and (4) maxillary anterior extrusion and occlusal refinement.

**Figure 7:**
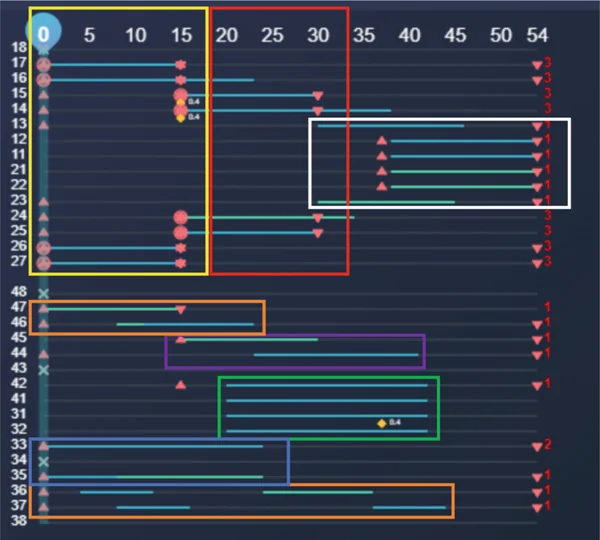
Staging of the first set of appliances. Yellow box shows Steps 1–15, vertical intrusion of upper molars. Red box shows Steps 16–30, vertical intrusion of the upper premolars. White box shows Steps 38–54, extrusion of the upper anterior teeth. Orange box shows Steps 0–45, move the right mandibular molar mesially and the left mandibular molar distally. Blue box shows Steps 1–25, adjust the torsion of the dental axes of the left canine and premolars. Purple box shows Steps 15–40, adjust the torsion of the dental axis of the right premolar. Green box shows Steps 20–42, leveling of mandibular anterior teeth.

**Figure 8:**
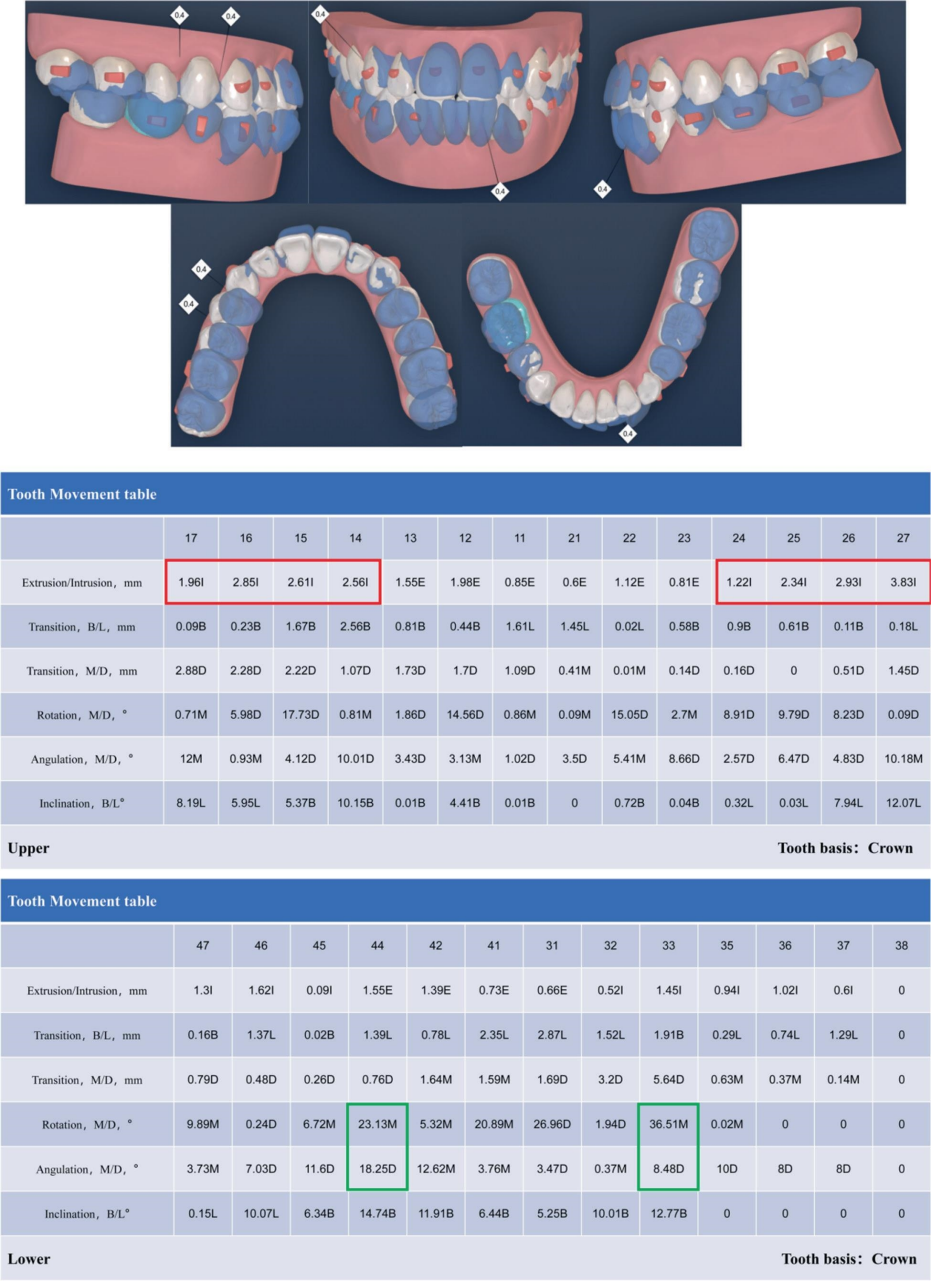
Smartee software plan and tooth movement table of the first set of aligners. Red boxes highlight the designed extrusion and maxillary posterior teeth intrusion. Green boxes indicate the designed torque movement of the right premolar and tipping movement of the left canine on the right side of the mandible.

The comprehensive treatment plan was executed following this staged protocol, as illustrated in the staging diagram (Figure 7). Each phase was meticulously planned using Smartee treatment planning software, which generated detailed movement protocols and force vectors to optimize biomechanical control (Figure 8).

#### 2.4.1. Phase 1: Maxillary Molar Intrusion (Steps 1–15)

**Figure 9:**
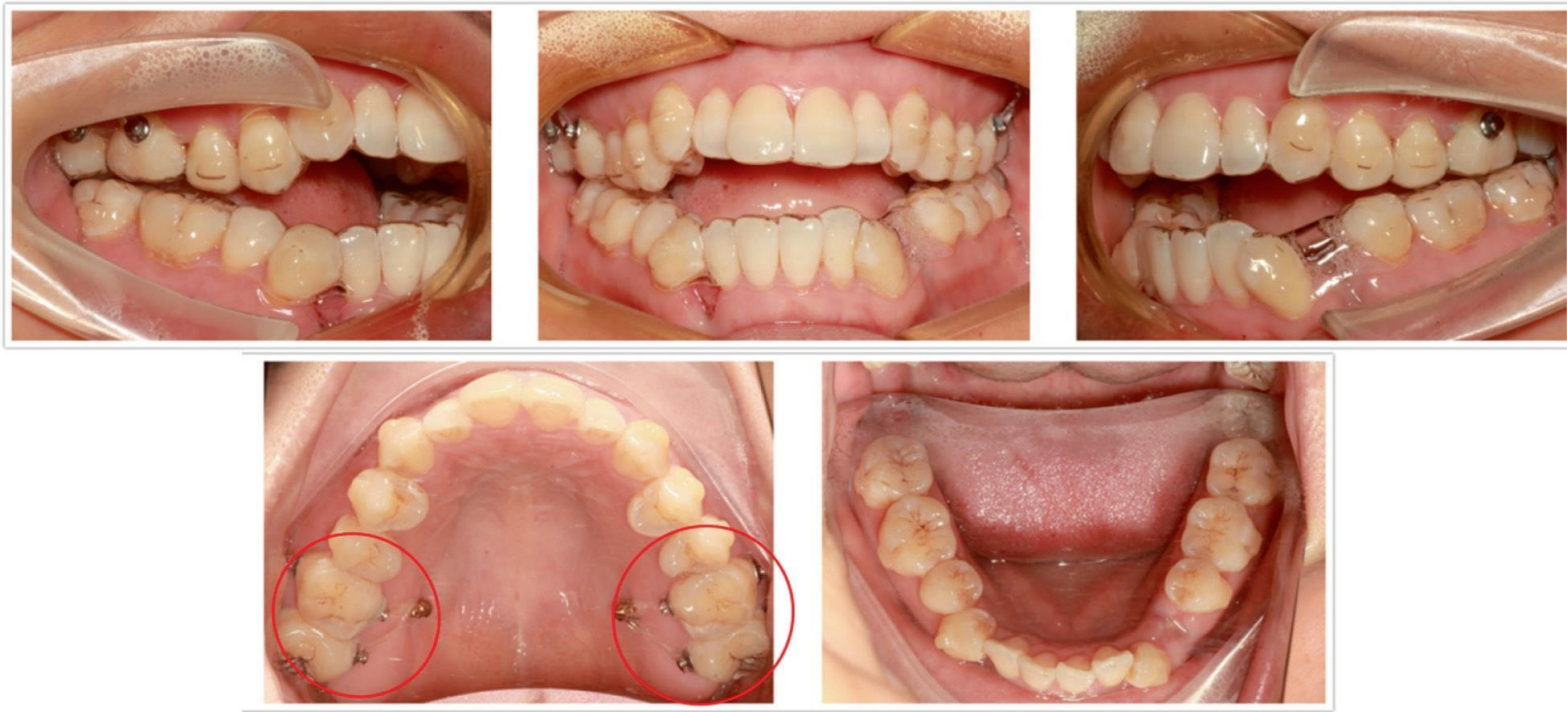
Treatment Process 1. Remove 34 and 43, and place one mini‐implant screw (a total of 4 screws) on the buccal–palatal side between the first and second molars on both sides of the upper jaw. Bond the lingual buttons to the gingival margin on the buccal–palatal side of the first and second molars and utilize the implant screws to depress the molars on both sides.

The initial phase established vertical control in the maxillary posterior region through strategic molar intrusion. Extractions of Teeth 34 and 43 were performed first under local anesthesia. Mini‐implant placement was carried out approximately 2 weeks later, after initial mucosal healing at the extraction sites had been confirmed clinically. The first set of aligners was delivered at the same appointment as miniscrew placement so that the intrusive elastic protocol and aligner therapy commenced simultaneously. Four self‐drilling titanium mini‐implants were placed in the maxillary posterior region—two buccal and two palatal, positioned between the first and second molars bilaterally (Figure 9).

Lingual buttons were bonded near the gingival margin on both buccal and palatal surfaces of the maxillary first and second molars to serve as elastic attachment points. Intrusive forces were delivered using elastic bands (1/4 inch, 3.5 oz) connecting the implants to the buttons, creating a controlled system for vertical displacement. This dual‐anchor approach enabled optimal force distribution, minimizing unwanted tilting while achieving predictable bodily intrusion.

#### 2.4.2. Phase 2: Maxillary Premolar Intrusion (Steps 16–30)

**Figure 10:**
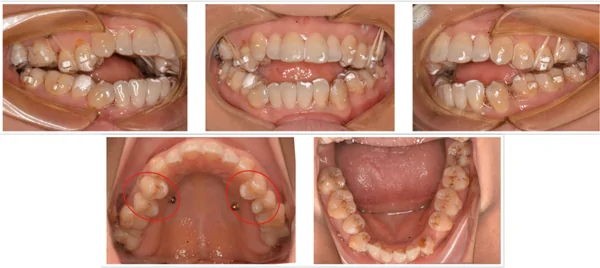
Treatment results of aligner No.15 at 8th month and treatment Process 2. After lowering the pressure in the region of the molars to the appropriate level, adjust the implant pins to the area of the premolars and reduce the pressure on both sides of the premolars.

**Figure 11:**
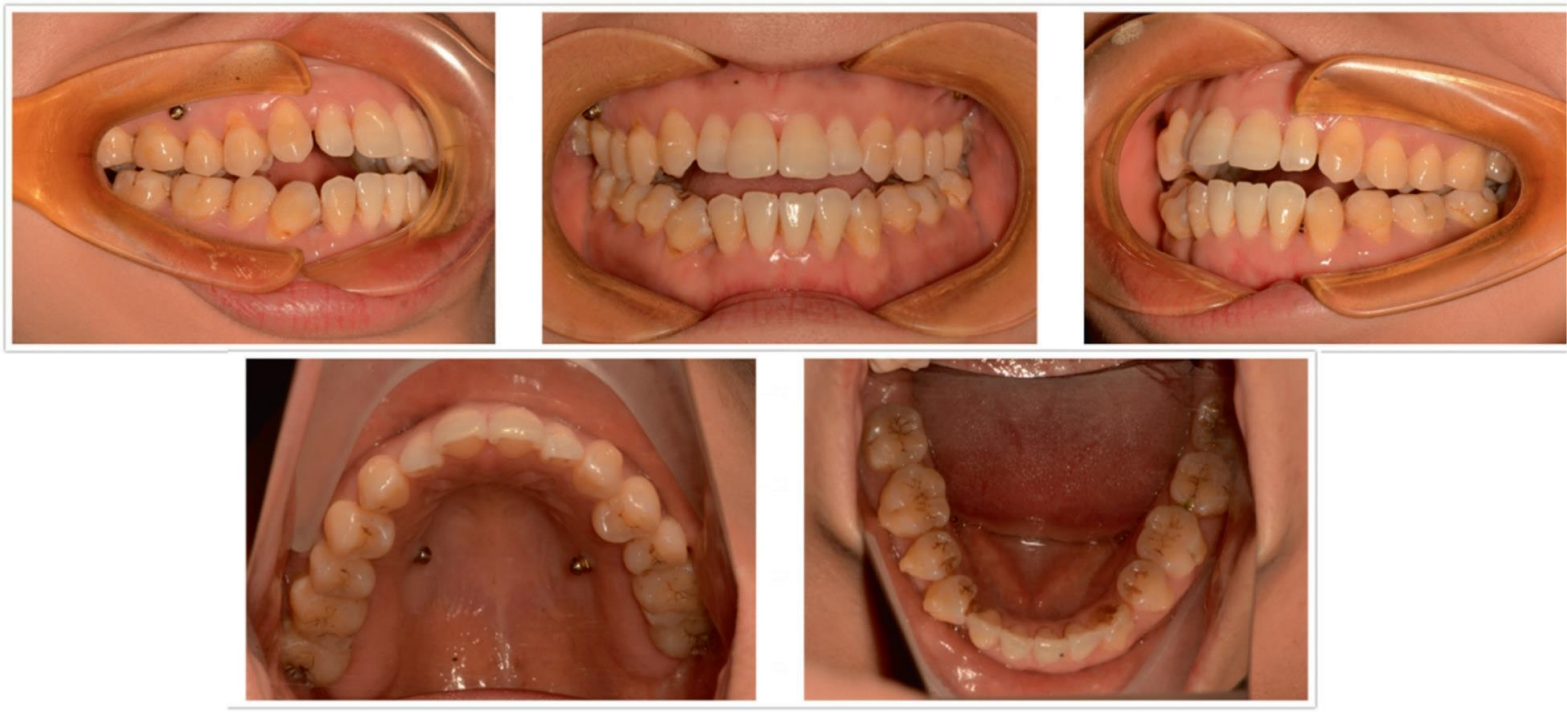
Treatment results of aligner No.30 at 16th month. Utilizing the orthopads effect of the invisible aligners and the implant screws to continuously lower the posterior teeth until the posterior teeth are gradually reconstructed.

Following successful molar intrusion at the 8‐month evaluation (Figure 10), the treatment progressed to premolar intrusion. Elastic bands (1/4 inch, 3.5 oz) were repositioned to connect the adjusted implant locations with attachment points on the maxillary premolars. The clear aligners (Steps 16–30) continued to provide the “bite block effect,” facilitating controlled vertical intrusion of both premolars while maintaining the achieved molar intrusion (Figure 11).

#### 2.4.3. Phase 3: Mandibular Anterior Alignment and Root Control (Steps 15–42)

**Figure 12:**
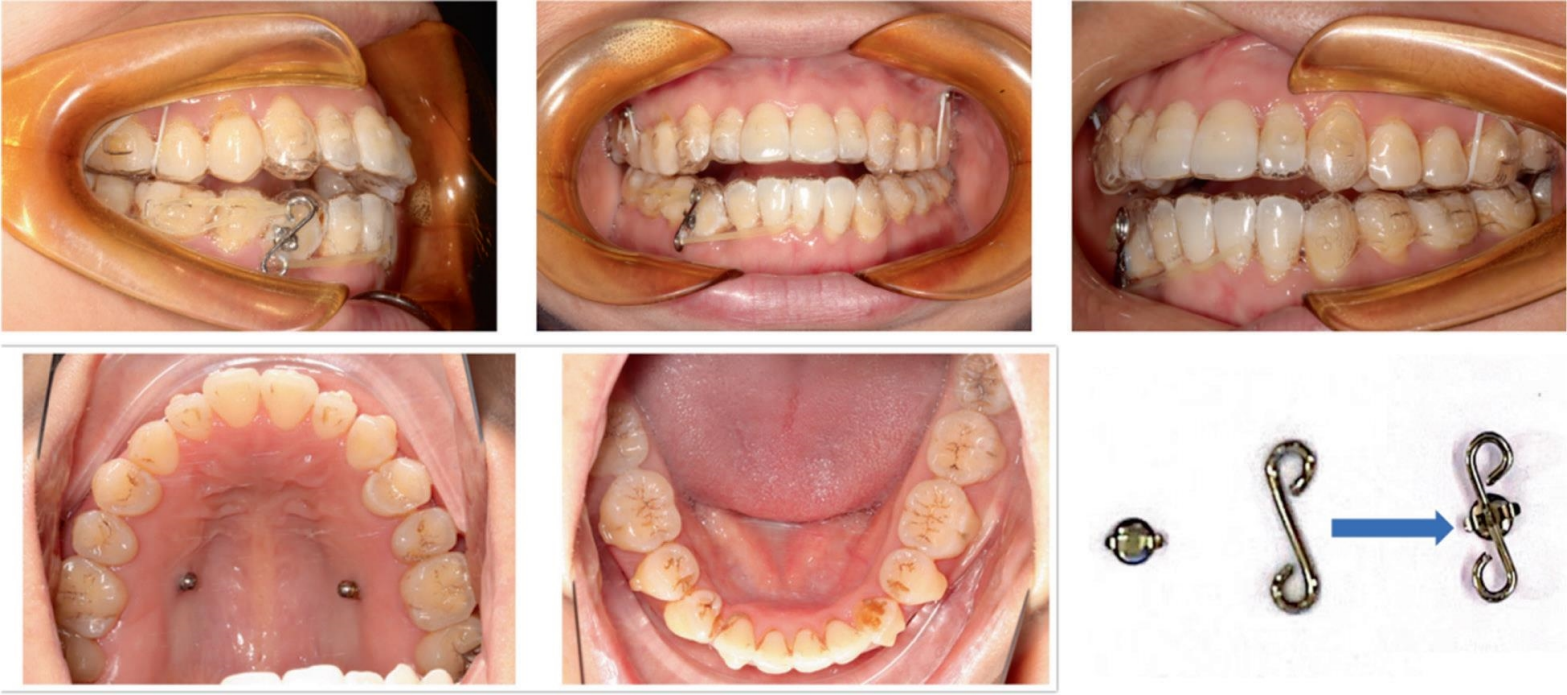
Treatment Process 3. The S‐shaped double‐arm root control attachment bonded to the buccal surface of Tooth 44 facilitates controlled root movement during mesial translation to substitute for extracted Tooth 43. The attachment engages in interactive traction between Tooth 44 and the mesial–distal walls of the aligner, creating controlled torque for root uprighting. In contrast, Tooth 33 undergoes tipping movement without specialized attachments. Vertical traction is applied to Teeth 13 and 23.

This phase addressed the correction of severely tilted mandibular teeth, with emphasis on axial inclination. Custom S‐shaped double‐arm root control attachments were bonded to Tooth 44 to guide its controlled mesial translation into the extraction space of Tooth 43 while preventing excessive mesial crown tipping during this movement (Figure 12).

**Figure 13:**
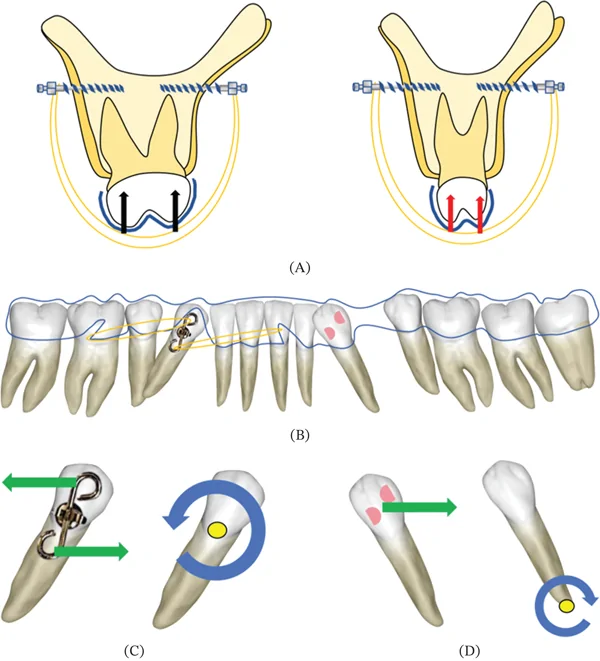
Biomechanical analysis. (A) Using invisible aligners to vertically intrude molars and premolars assisted by buccal and palatal implants. (B) S‐shaped double‐arm root control attachment to adjust mandibular Tooth 44 torsion during mesial movement to substitute for extracted tooth 43. (C) The force and center of rotation of Tooth 44 (right mandibular first premolar) showing controlled root movement. (D) The force and center of rotation of Tooth 33 (left mandibular canine) showing tipping movement without root control attachments. Blue lines indicate invisible aligners, yellow lines indicate rubber bands, black arrows indicate molar vertical reduction, red arrows indicate premolar vertical reduction, green arrows indicate the direction of force, blue arrows indicate the direction of rotation, and yellow dots indicate the center of rotation.

The SRCA consists of two curvilinear composite resin arms extending in opposite mesiodistal directions from a central bonding pad, forming an S‐shaped profile when viewed from the buccal aspect (Figure 12; Figure 13B). Each arm, approximately 2 mm in length, engages the inner surface of the corresponding aligner cavity wall when the aligner is fully seated: the mesial arm contacts the mesial wall, and the distal arm contacts the distal wall. This dual‐wall engagement creates a force couple at the crown level: the mesial arm resists mesial crown tipping, whereas the distal arm transmits a torquing moment toward the root apex. The critical biomechanical consequence is a shift in the effective center of rotation—away from the region near the root apex (as occurs with conventional single‐surface attachments, or with no attachment at all, both of which generate predominantly tipping moments) and toward the center of resistance of the tooth (approximately one‐third of the root length from the apex). This redistribution substantially increases the moment‐to‐force (M/F) ratio at the root, enabling more controlled bodily translation with reduced total crown displacement (Figure 13C). The attachment was fabricated chairside from light‐cure flowable composite resin and bonded to the mid‐buccal surface of tooth 44 using standard phosphoric‐acid etch and single‐component bonding resin. The total attachment height from bonding pad to arm tip was approximately 3–4 mm, a dimension designed to remain within the available occlusal clearance. In contrast to conventional rectangular attachments, which primarily enhance aligner grip for tipping movements, the S‐shaped dual‐arm configuration was specifically engineered to address root inclination correction during translational tooth movement.

Concurrent bilateral elastic traction (5/16 inch, 3.5 oz) assisted in mandibular alignment and correction of the axial inclination of Tooth 44, which was being moved mesially to substitute for the extracted right canine. In contrast, Tooth 33 underwent planned tipping movement without specialized attachments (Steps 1–25), as its position permitted effective correction through controlled crown movement. This differential approach—root control for Tooth 44 versus tipping for Tooth 33—optimized efficiency by addressing each tooth′s specific requirements (Figure 13C,D). Comprehensive mandibular anterior leveling (Steps 20–42) proceeded while maintaining the established posterior intrusion.

#### 2.4.4. Phase 4: Maxillary Anterior Extrusion and Final Refinement (Steps 38–54)

The final phase focused on AOB closure through controlled extrusion of maxillary anterior teeth. Based on the Smartee treatment planning data for Steps 38–54, the total planned extrusion for the central and lateral incisors (Teeth 11, 12, 21, and 22) ranged from approximately 0.6 to 2.0 mm, whereas Teeth 13 and 23 underwent 1.5 mm of extrusion each, facilitated by vertical elastic traction (5/16 inch, 3.5 oz) applied between the miniscrew heads and buccal hooks bonded to the canines.

The esthetic implications of anterior extrusion were evaluated prospectively during treatment planning. Given the patient′s preexisting maxillary incisor retroclination (U1‐SN: 100.91°) and reduced incisor display at rest, controlled extrusion of the upper anterior teeth was anticipated to improve the smile arc and increase incisor display during animation—both identified as esthetic improvement targets at the initial consultation. Digital smile simulation within the Smartee planning software confirmed that the planned extrusion magnitude would not produce excessive gingival display (gummy smile), and the posttreatment simulation demonstrated harmonious smile esthetics consistent with the patient′s facial proportions. Posttreatment clinical examination confirmed that incisor display and smile arc were within esthetically acceptable parameters, consistent with the planned outcome. Vertical traction was applied to Teeth 13 and 23, whereas aligners delivered extrusive forces to the maxillary incisors.

### 2.5. Treatment Monitoring and Adjustments

**Figure 14:**
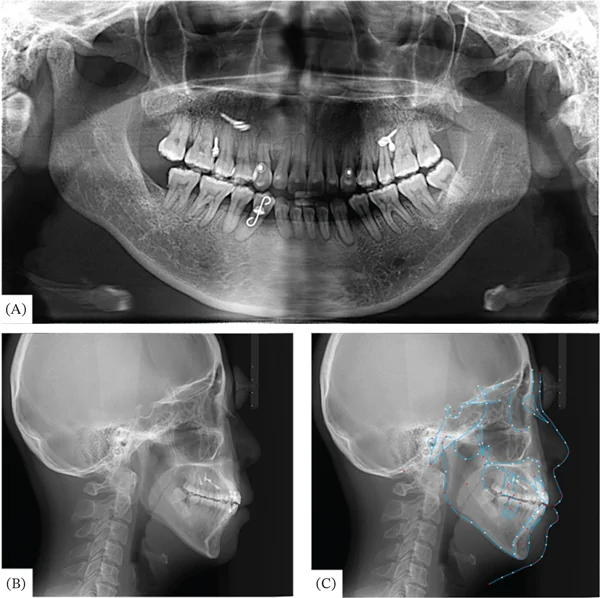
On‐treatment radiographs. (A) Panoramic radiograph, (B) lateral cephalogram, and (C) lateral cephalogram tracing.

The clear aligners used in this case were the Zhengya Classic Series (Zhengya Orthodontic Technology Co. Ltd., China), fabricated from a 1.0‐mm‐thick proprietary single‐layer thermoplastic membrane. All aligners were manufactured at the Zhengya factory using digital models and the treatment plan designed in Smartee planning software. Each aligner step was programmed with a maximum tooth movement of ≤ 0.2 mm per step, in accordance with the manufacturer′s protocol for controlled incremental activation. Throughout the 30‐month treatment period, aligners were replaced at 14‐day intervals with regular monitoring appointments, a schedule consistent with established guidelines for periodontal ligament remodeling and biologically safe force application. Given the patient′s preexisting periodontal condition, close interdisciplinary collaboration with the periodontology department was maintained throughout the entire treatment period. Pretreatment periodontal assessment, conducted prior to orthodontic commencement, recorded the following baseline parameters: mean full‐mouth probing depth (PD), 3.2 mm; mean clinical attachment level (CAL), 3.8 mm at the most severely affected sites (Teeth 16,26,36,46); full‐mouth bleeding score (FMBS), 28%; and radiographic bone loss of approximately 15% of root length at sites of greatest alveolar involvement (Figure 3A). Active periodontal therapy, including scaling and root planing was completed, and periodontal stability was confirmed before orthodontic treatment commenced. The patient received three‐monthly professional periodontal maintenance throughout orthodontic treatment. Midtreatment periodontal assessment at 16 months recorded mean PD of 2.8 mm and FMBS of 18%, with CALs remaining stable compared to baseline. Under guidance from periodontists, the treatment plan incorporated light continuous forces to minimize adverse effects on the periodontal tissues. Progress was assessed through clinical examination and periodic radiographic evaluation. The midtreatment radiographic assessment (Figure 14) confirmed appropriate root positioning with no evidence of significant adverse periodontal effects.

### 2.6. Treatment Result

**Figure 15:**
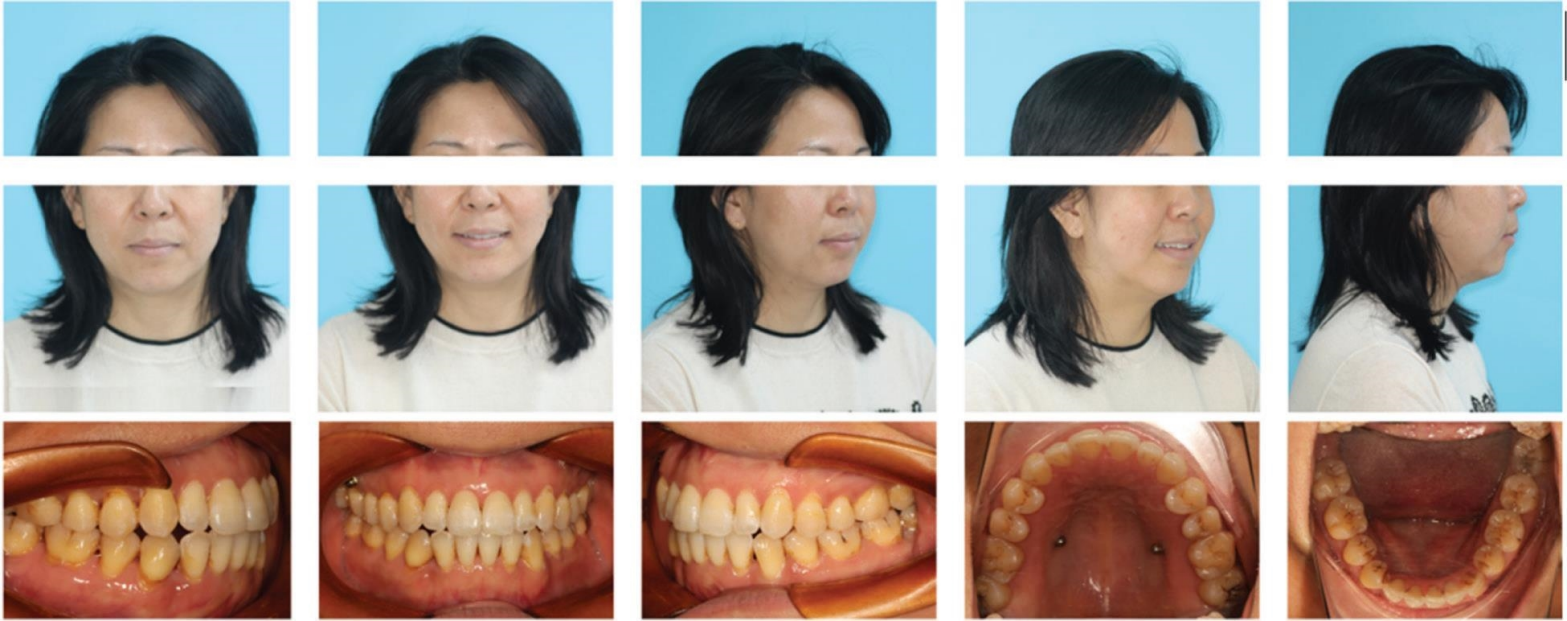
Final facial and intraoral photographs.

Evaluation after 30 months of systematic treatment demonstrated comprehensive correction of the AOB with significant improvement in both occlusal function and facial esthetics (Figure 15). The therapeutic outcomes included establishment of normal anterior overbite and overjet relationships alongside complete resolution of dental crowding in both arches. The achieved posterior tooth intrusion resulted in beneficial counterclockwise rotation of the mandible, contributing to improved facial proportions. Occlusal relationships showed marked improvement, with the left canine achieving a neutral Class I relationship, whereas the right canine maintained a slight Class II relationship. Bilateral molars retained functionally acceptable mesial relationships, and dental midlines were properly aligned.

**Figure 16:**
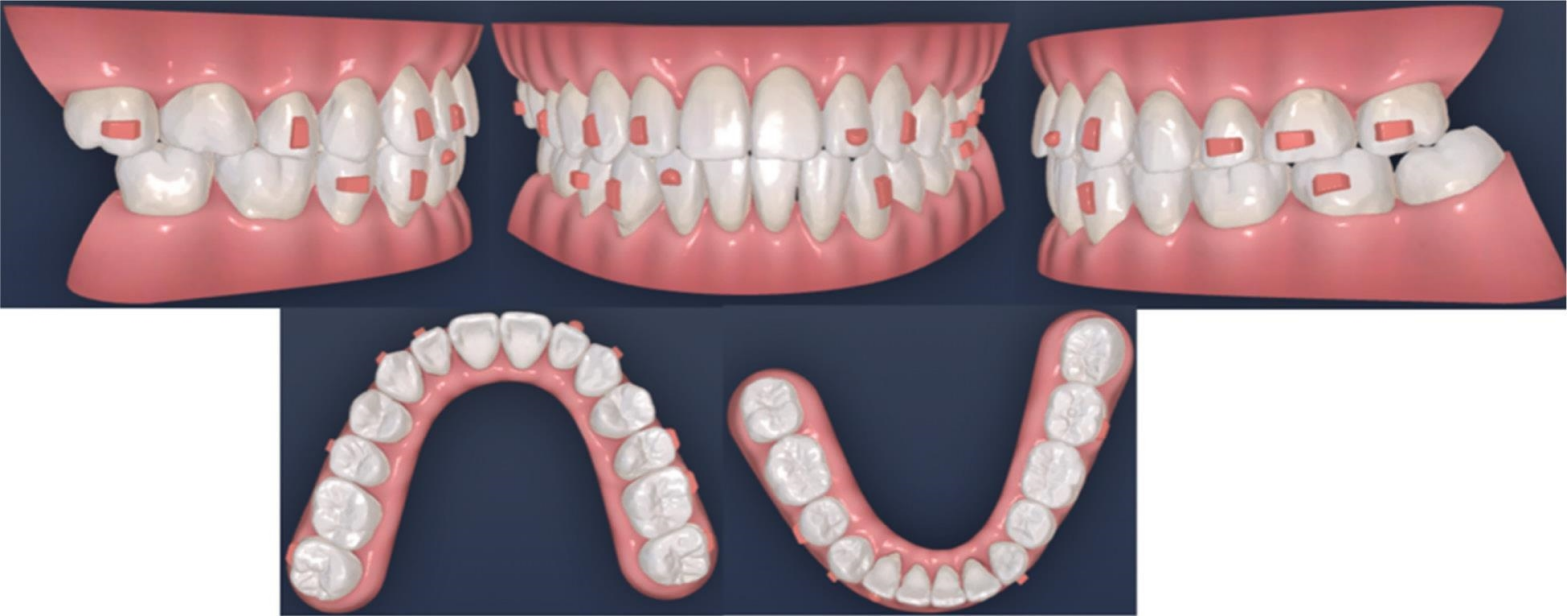
Posttreatment digital model in Smartee software.

Posttreatment digital model analysis confirmed satisfactory arch form and interarch coordination (Figure 16). However, several treatment limitations should be acknowledged. Regarding Tooth 44, the incomplete root uprighting to ideal angulation was primarily due to the patient′s personal decision to conclude active treatment, as she expressed satisfaction with the achieved esthetic and functional outcomes and requested progression to the retention phase. It should be noted that with additional treatment time, complete root parallelism could have been attained. The right posterior intercuspation showed functionally acceptable though not ideal occlusal contacts, representing a necessary compromise between AOB correction and posterior stability.

**Figure 17:**
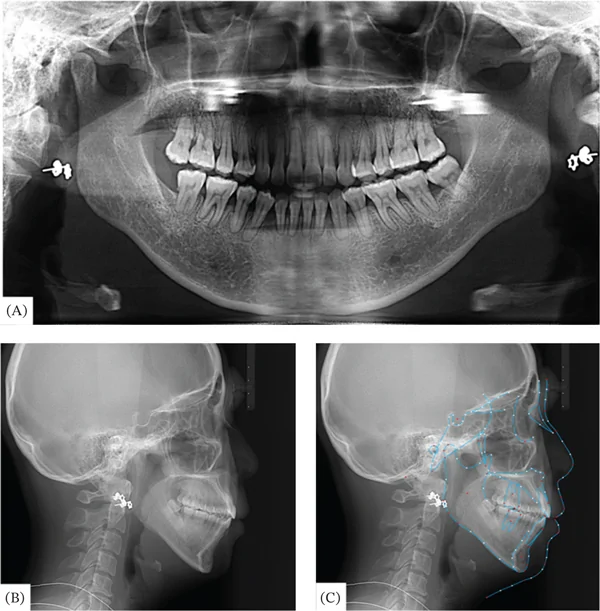
Posttreatment radiographs. (A) Panoramic radiograph, (B) lateral cephalogram, and (C) lateral cephalogram tracing.

Considering the patient′s preexisting periodontal condition, interdisciplinary collaboration with the periodontology department was maintained throughout treatment, and the patient received ongoing periodontal therapy and maintenance care. Posttreatment periodontal evaluation documented mean full‐mouth PD of 3.0 mm, mean CAL of 4.2 mm (indicating mild loss of 0.4 mm relative to pretreatment baseline), and FMBS of 15%. Radiographic analysis of the posttreatment panoramic radiograph (Figure 17A) identified mild alveolar bone loss at Teeth 16 and 26, with estimated crestal bone reduction of approximately 18% of root length and root shortening of approximately 1.2 mm at the sites subjected to the greatest intrusive forces during posterior tooth movement. Despite consistent application of light forces and regular periodontal maintenance, these findings remained within the range deemed clinically acceptable given the extent and duration of treatment, and no acute periodontal episodes requiring cessation of tooth movement occurred throughout treatment. This outcome underscores the biological challenges inherent in managing complex orthodontic tooth movements in a periodontally compromised patient, and highlights the importance of careful risk–benefit assessment and close interdisciplinary monitoring.

**Figure 18:**
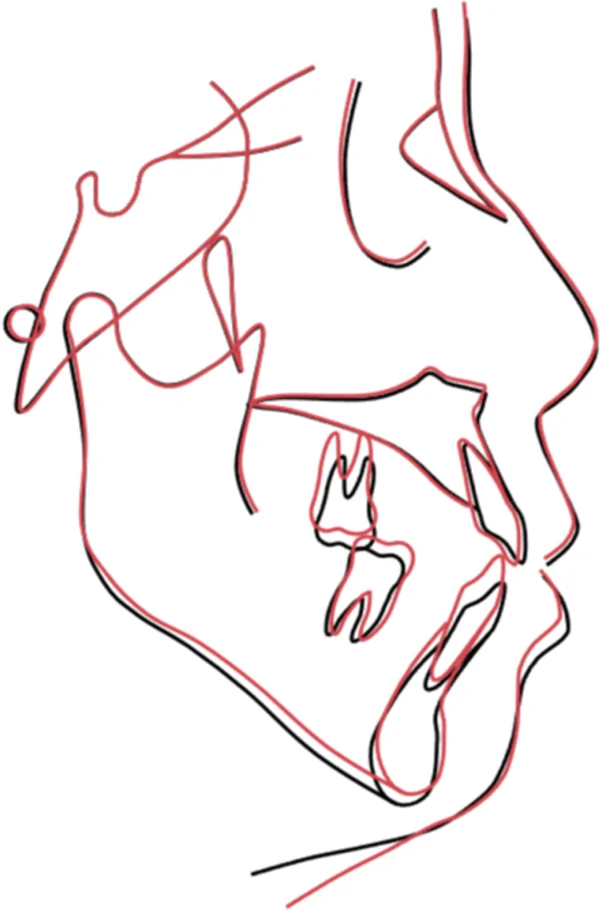
Superimposed cephalometric tracings at pretreatment (black) and posttreatment (red).

**Figure 19:**
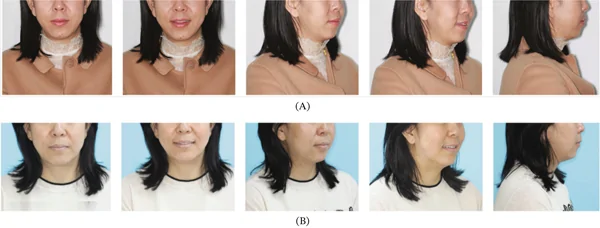
Comparison of appearance: a counterclockwise rotation of the mandible. (A) pretreatment and (B) posttreatment.

Superimposed cephalometric tracings (Figure 18) were constructed using the anterior cranial base (SN plane registered at Sella) as the primary reference structure, the standard method for longitudinal overall skeletal evaluation. The overlays demonstrated systematic posterior tooth intrusion, with the reduction in SN‐MP angle (50.63°–49.44°) reflecting a combination of true dentoalveolar molar intrusion and secondary counterclockwise mandibular autorotation following reduction of posterior occlusal height. Cephalometric analysis further demonstrated favorable skeletal changes: the SNB angle increased from 75.61° to 76.02° and the ANB angle decreased from 6.49° to 5.73° (Figure 17B,C; Table 1). Superimposed cephalometric tracings clearly illustrated the systematic posterior tooth intrusion, controlled anterior tooth positioning, and significant enhancement of the facial profile. The pretreatment convex facial profile with excessive lower facial height was substantially improved, with elimination of lip incompetence and achievement of enhanced facial harmony (Figure 19).

**Table 1:** Pretreatment and posttreatment cephalometric analysis.

Measurement	Norm ± SD	Pretreatment	Posttreatment
SNA	83 ± 4	82.1	81.75
SNB	80 ± 3	75.61	76.02
ANB	3 ± 2	6.49	5.73
SN‐MP	33 ± 4	50.63	49.44
S‐Go/N‐Me(%)	66 ± 4	56.48	56.21
U1‐L1	127 ± 9	118.6	127.8
U1‐SN	105 ± 6	100.9	98.58
U1‐NA(mm)	4 ± 2	5.55	4.56
IMPA	92.6 ± 7	89.82	84.15
L1‐NB(mm)	6.7 ± 2.1	11.49	10.3
UL‐EP(mm)	−1.4 ± 0.9	3.84	2.76
LL‐EP(mm)	0.6 ± 0.9	5.93	3.48
Z angle	71 ± 5	66.39	67.99

### 2.7. Retention and Follow‐Up

**Figure 20:**
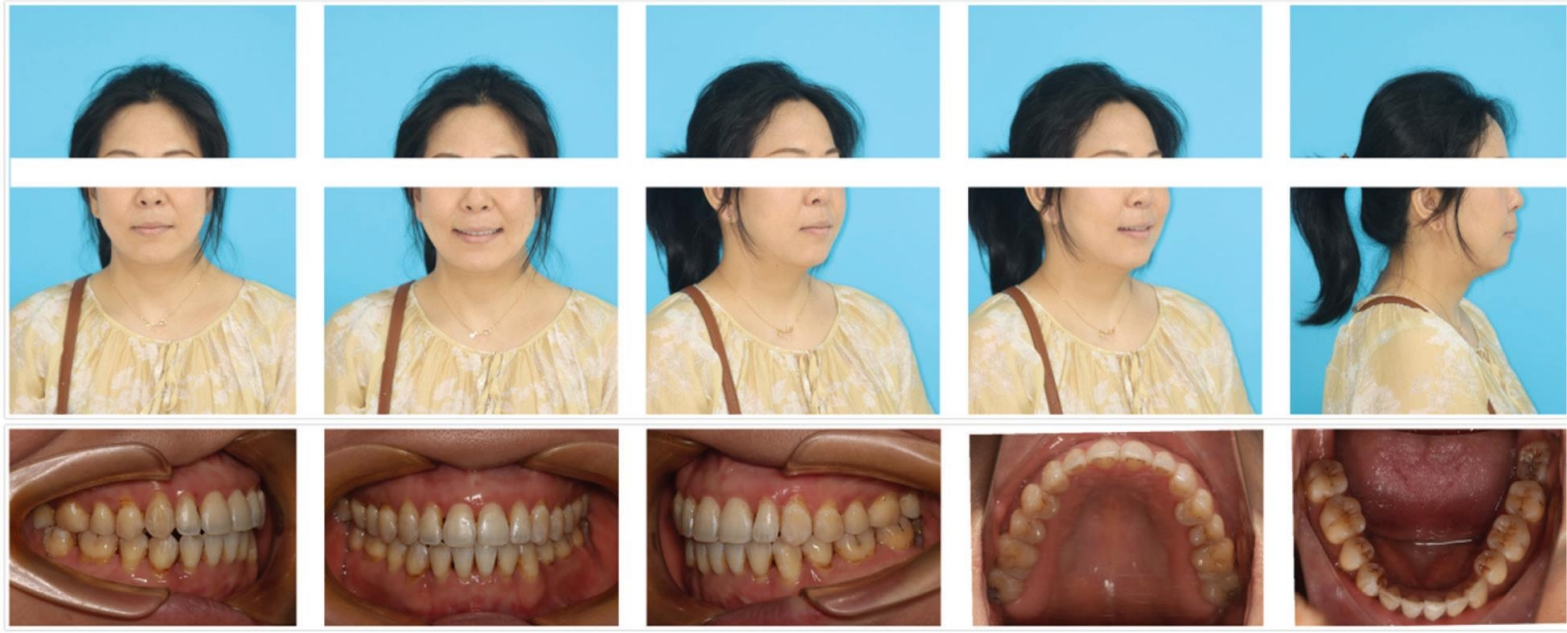
13‐month post‐retention facial and intraoral photographs.

Upon completion of active treatment, both arches were retained using conventional vacuum‐formed transparent removable retainers (full‐coverage and tooth‐supported). No posterior occlusal coverage or bite pads were incorporated; posterior vertical stability was maintained through established interdigitation and periodontal adaptation. The patient was instructed to wear retainers full‐time (approximately 22 h/day) for the first 12 months, after which a transition to nighttime‐only wear is planned. Follow‐up appointments are scheduled every 6 months. At 13 months postretention, clinical evaluation (Figure 20) confirmed excellent stability of all treatment outcomes, with no significant relapse of the AOB, overbite, or overjet relationships.

## 3. Discussion

The present case demonstrates the effective management of a complex AOB through an integrated approach combining implant‐assisted CAT with specialized root control attachments. This protocol successfully addressed the primary etiological factors of excessive posterior vertical development and severe dental crowding while accommodating the patient′s strong preference for an esthetic and nonsurgical treatment modality.

CAT offers distinct advantages for vertical control in AOB correction. The inherent “bite block effect” provides continuous vertical force on posterior teeth, potentially facilitating intrusion [10, 13]. However, substantial posterior intrusion remains challenging with aligners alone due to material limitations and inadequate force expression [14, 15]. The strategic placement of mini‐implants on both buccal and palatal aspects in this case created a stable anchorage system that overcame these limitations (Figure 13A) [16]. This bilateral approach enabled controlled vertical displacement of the entire molar complex while minimizing undesirable tipping movements [17, 18].

The sequential intrusion protocol—addressing molars first (Steps 1–15) followed by premolars (Steps 16–30)—proved biomechanically advantageous. This staging created posterior disocclusion, reducing resistance to subsequent intrusion and allowing biological adaptation of periodontal tissues [19, 20]. The progressive reduction in posterior occlusal contacts facilitated more efficient force transmission during the premolar intrusion phase, enhancing treatment predictability [21, 22].

The likelihood and extent of mandibular counterclockwise rotation achievable through nonsurgical posterior intrusion in adult patients warrants careful consideration, as it directly informs appropriate case selection. Unlike growing patients in whom true skeletal rotation is achievable through growth modification, adult patients have completed craniofacial development; changes in mandibular plane angle are predominantly dentoalveolar rather than truly skeletal in nature. In the present case, the observed reduction in SN‐MP from 50.63° to 49.44° reflects a combination of true dentoalveolar molar intrusion and secondary counterclockwise mandibular autorotation. As posterior occlusal height is reduced through molar and premolar intrusion, the mandible rotates counterclockwise around the condylar fulcrum as the condyle reseats in the glenoid fossa. The magnitude of achievable rotation in adults is constrained by several factors: (1) increased cortical bone density limits the rate and extent of alveolar remodeling and therefore the maximum achievable intrusion; (2) established masticatory muscle resting lengths and tonicity resist sustained changes in vertical facial dimension; (3) condylar remodeling and adaptive capacity are reduced compared to growing patients; and (4) available root length and alveolar bone height impose a biological ceiling on intrusion depth, particularly in periodontally compromised patients. Collectively, these factors mean that clinically achievable nonsurgical CCW rotation in adults typically produces SN‐MP reductions of 1°–3°, consistent with the 1.19° reduction observed in this case rather than the broader skeletal changes achievable surgically.

This treatment approach is most appropriate when the hyperdivergent pattern is of mild‐to‐moderate severity (SN‐MP 40°–55°), the AOB is predominantly dentoalveolar in origin and does not exceed 6–8 mm, posterior alveolar bone height is sufficient to accommodate multimillimeter molar intrusion under TAD anchorage, no severe transverse or sagittal jaw discrepancy is present, and periodontal health is compatible with extended orthodontic tooth movement. Orthognathic surgery should be considered the treatment of choice when the vertical discrepancy is primarily skeletal, with true posterior alveolar excess confirmed on three‐dimensional imaging; the ANB exceeds 8°–10° with significant sagittal jaw discrepancy; the AOB exceeds 8 mm or is associated with pronounced mandibular plane steepness (SN‐MP > 55°); unfavorable muscle patterns predispose to relapse; or when the degree of posterior intrusion required would compromise root integrity or periodontal health to an unacceptable degree. In the present case, the nonsurgical approach was justified by the predominantly dentoalveolar open bite etiology, adequate posterior bone height, an objective CAT‐CAT score within the manageable range, and the patient′s informed refusal of surgical intervention—a decision subsequently validated by the 13‐month postretention stability data.

Beyond intrusion challenges, the management of severely inclined teeth represented another significant biomechanical hurdle. The S‐shaped double‐arm root control attachments provided an effective solution by fundamentally altering the force system applied to Tooth 44. Traditional aligner therapy often struggles with adequate root torque because conventional attachments, whether rectangular, beveled, or optimized, primarily generate forces that keep the center of rotation near the root apex, resulting in predominantly tipping movements. The S‐shaped design overcomes this limitation by creating two simultaneous force application points on opposite walls of the aligner cavity, the mesial arm and distal arm act in concert to produce a force couple whose net effect shifts the center of rotation from the root apex toward the center of resistance, substantially increasing the M/F ratio without requiring an increase in total applied force. This mechanism enabled predictable bodily mesial translation of Tooth 44, including controlled root movement, into the space of the extracted canine while avoiding the excessive crown tipping and root prominence that would result from conventional tipping mechanics in a severely inclined tooth.

The differential application of attachments based on individual tooth requirements demonstrated strategic biomechanical planning. For Tooth 44, which required controlled root movement during mesial translation to substitute for the extracted canine, the S‐shaped attachment facilitated bodily movement with minimal crown displacement (Figure 13C). In contrast, Tooth 33 responded adequately to conventional tipping mechanics for rotation correction and alignment (Figure 13D). This case‐specific approach optimized treatment efficiency and highlighted the importance of customized biomechanical solutions in complex cases [23, 24].

Several limitations warrant consideration in this case. The patient′s preexisting periodontal condition necessitated ongoing interdisciplinary management with periodontists throughout treatment. Despite consistent application of light forces and regular periodontal maintenance, some alveolar bone loss and root resorption occurred, particularly in areas subjected to intrusive forces. This underscores the biological challenges of complex orthodontic movements in compromised periodontal environments and highlights the importance of careful risk‐benefit assessment and patient education.

Furthermore, the incomplete root uprighting of Tooth 44 reflects the balance between ideal treatment goals and patient satisfaction in clinical practice. Although additional treatment time could have achieved better root parallelism, the patient′s decision to conclude active treatment based on her satisfaction with the esthetic and functional outcomes represents an important aspect of patient‐centered care.

The 13‐month postretention follow‐up documented in Section 2.7 (Figure 20), demonstrating stable overbite and overjet with no significant relapse of the AOB, provides preliminary evidence supporting the adequacy of this nonsurgical protocol for appropriately selected adult patients, although longer‐term follow‐up data would further strengthen conclusions regarding stability.

## 4. Conclusion

This case report illustrates the successful correction of a complex anterior open bite malocclusion using a meticulously planned protocol of implant‐assisted CAT augmented by specialized root control attachments. The strategic combination of TADs with clear aligners expands the therapeutic possibilities for managing challenging malocclusions while maintaining patient comfort and esthetic demands. The integrated approach described—incorporating staged posterior intrusion, customized attachments for root control, and interdisciplinary periodontal management—provides a valuable framework for addressing similar complex cases in clinical practice.

## Author Contributions

Y.C. and Y.X. contributed to the drafting of the manuscript. Y.C., F.Q., C.L., and X.S. contributed to the dental examination and dental treatment of the patient. Q.J., Y.C., and Y.X. contributed to the conception and design of the study.

## Funding

This work was supported by the Guangxi Medical and Health Appropriate Technology Development and Application Project, No.S2022153; the Guangxi Zhuang Autonomous Region Health Commission self‐funded research project, No.Z‐C20231971; Project to improve basic research ability of young and middle‐aged teachers in Guangxi colleges and universities, No.2024KY0499; and the Guilin Science Research and Technology Development Program, No.20230135‐5‐1.

## Disclosure

All authors approved the version submitted for publication. All authors read and approved the final manuscript.

## Consent

Written informed consent was obtained from the patient prior to treatment initiation, covering orthodontic treatment procedures, risks and benefits, and the use of de‐identified clinical photographs, radiographs, and case data for academic publication. The consent document is retained in the patient′s clinical record in accordance with the institutional regulations of the Affiliated Stomatology Hospital of Guilin Medical University.

## Conflicts of Interest

The authors declare no conflicts of interest.

## Data Availability

The data that support the findings of this study are available from the corresponding author upon reasonable request.
